# Supplementary material for: Impacts of parental age and inbreeding on fitness in a wild insect
Source: PLoS Biol. 2026 Mar 10;24(3):e3003713. doi: 10.1371/journal.pbio.3003713 (PMC12991364; doi:10.1371/journal.pbio.3003713)
Supplement: Attachment — Submitted filename: PLOS Biology response to reviewers final.docx [file pbio.3003713.s004.docx]

Thank you very much for the time that the reviewers have put into our manuscript. We were very encouraged by the reviewers’ comments and there are a lot of useful points which have led to modifications of our design, and to how we frame the study. The major aims and expected outcomes have not been affected by these improvements, and our revised power analysis indicates that we will still have power to detect the effects we are interested in, even when placing more emphasis on an among-individuals comparison in order to increase range of parental ages we study. Please find below descriptions of the changes that we’ve made in response to the reviewers’ comments. Reviewer comments are in red with our replies in black below.

Rev. 1:
The proposed experiment addresses a very interesting and timely question: how parental age at breeding affects offspring phenotype and performance, and whether such effects are modulated by the ambient environment that parents experience. The effects of senescence on offspring remain poorly understood, and represent a fascinating example of nongenetic inheritance. This study is especially valuable in that it aims to investigate effects of parental age at breeding in a natural population. While a few studies have investigated such effects in the wild, very few have manipulated parental age at breeding and none have also manipulated the parental environment.

Thanks, we are very excited about the study

Nonetheless, I have a few concerns about the design of the proposed study.

Good.

Major comments:

I have a couple of concerns in relation to the proposed experiment design.

First, I think it's problematic to assume that the temperature treatment will affect the offspring only via effects on maternal ageing. The authors refer to individuals that are chronologically old but kept at the lower temperature as "biologically young." This is surely an assumption.

Yes, this is a sensible point; if we understand correctly, the reviewer is pointing out that the temperature treatment might cause a parental effect not only because of an influence that relates to ageing, but might also have effects that would not normally be thought of as being part of ageing. We have modified our text extensively to address this point:

An aim of our study is to separate endogenous processes which might cause deterioration in individuals and subsequently affect their offspring, from exogenous processes that might have the same effect. For instance, cellular oxidative stress will be dependent upon temperature via metabolic rate, whereas DNA damage caused by UV radiation, or physical damage caused by interactions with the environment are expected to accumulate as a result of the passage of time and hence are not necessarily temperature dependent. Also, in general, senescence has tended to be studied by simply measuring chronological age, and this is surely not an appropriate approach in ectotherms where development times and lifespans are invariably extremely temperature dependent. We characterised endogenous effects as ‘biological ageing’ and exogenous effects as ‘chronological ageing’. However, we agree with the thrust of the reviewer’s point, that this terminology is liable to be interpreted in different ways by different readers, and also that it hides an assumption which may not be justified. We have removed the term ‘biological ageing’ and instead refer to endogenous and exogenous effects and to physiological ageing. We have also modified the legend to Table 1 so that it explains the physiological age treatment directly.

More important: on reflection, in our design, by bringing animals into the lab we remove the vast majority of exogenous stresses: lab crickets are not subject to interactions with other animals, UV is severely reduced and so on. Under these circumstances chronological ageing is likely to be equivalent to purely endogenous effects (which we previously called biological ageing). We have modified our design so that we will mate crickets at a range of ages creating variation in parental age. We will also exploit variation in age of maturity created by our two grandparental family rearing environments so that we have a range of seasonal laying dates that are not confounded with parental age. This allows us to remove the additional control group in our previous design. It also means that we are less reliant on comparing offspring from the same individuals when they are young and old in order to test our hypotheses. We will retain the within-individual comparisons but we have reduced our expectations of how many individuals will successfully re-mate and produce offspring in the light of studies we carried out this spring. The revised design is shown in Figure 1. We have also modified our power analysis so that we include the larger sample sizes that we’ll now have in the 4 main treatment groups (factorial young/old and inbred/outbred); we also made more conservative assumptions about how much additional power we will get from the within-individuals design, so the new power analysis shows both a purely among-individuals design and a weak improvement based on the within individual aspect of our design. I realise the above explanation is quite hard to follow (!) and I refer the reviewers to the revised ms which we hope is clear on these points.

It also looks like the design is oddly unbalanced: there are no O-Y-A or I-Y-A treatment combinations (Table 1), presumably because the authors don't see any reason to investigate effects of warm temperature on young-breeding crickets. But it's possible that ambient temperature would induce some kind of parental effect even in young crickets. It seems important to me to treat temperature as a factor independent of assumed effects on ageing, and include O/I - Y - A treatment combinations in the experiment. In other words, I would expose even the young-breeding crickets to both cool and warm temperature treatments, to see whether a brief exposure when young would still affect offspring phenotype
and performance.

This would indeed be an interesting avenue to explore. The omission of this treatment was not because we don’t see any reason to investigate it; we agree with the reviewer that it would be very interesting. We omitted the treatment because there is a limit to the number of adults in which we can measure fitness related traits in our meadow. We are already at this limit (~480), so adding additional treatments comes at the cost of reducing our power to investigate the treatments that we already have. Our power analysis indicates that we will have enough power with our current design but if we add new treatments to investigate the effects of temperature on young crickets as well as adults, we will seriously compromise the power we have to investigate the effects we are most interested in. A further point (although not the deciding factor) is that this study is already right at the limit of the work that we are able to carry out in terms of the person hours required in the breeding season. However, other readers will have the same thoughts as the reviewer, so in our revision, we have reduced our emphasis on the temperature manipulation in our design by creating a broader range of parental ages in the first mating that pairs engage in (with temperature standardised across pairs, but unmanipulated). Our plan is to use the discussion in the final manuscript to address the point raised by the reviewer (that temperature might affect ageing, but might also have other effects independent of ageing). It will be much easier to make this a cogent and comprehensible point once it is in the context of our results, whatever they may be.

Second, the experiment description is also unclear on a fundamental point: Will the temperature and age-at-breeding treatments be applied to females only, to both sexes together, or to each sex independently? How this is set up will determine whether the results can reveal whether effects are maternal or paternal, or depend on an interaction of maternal and paternal treatment. Ideally, females and males should be exposed to treatments independently, and then crossed in all combinations (full factorial design). That would allow the authors to determine effects of maternal treatments, paternal treatments, and maternal x paternal interactions. If this is not possible for practical reasons then the next best option would be to expose females to treatments and then pair all females with males of standard age and temperature treatment. That would allow the authors to test for maternal effects *per se*. Exposing both sexes together will make it impossible to determine whether effects
are maternal or paternal or depend on an interaction of the two.

As with the previous point, this is something that we agonised over in our original design meetings. We completely agree that the full factorial design would be the best. As the reviewer anticipates, this would create an enormous additional workload which we cannot sustain. Also, and more fundamentally, as per the previous point, adding more treatment groups reduces the power that we have to test our specific hypotheses. This is a very demanding study and we repeatedly had to make compromises on the breadth of the study in order to maximise power. This also affected our decision to age both males and females equally. As the reviewer anticipates, this means that we can measure parental effects overall, but cannot break them down into maternal and paternal effects. However, because our power is limited, rather than just ageing females as the reviewer suggests, we opted to age both parents because if they both have an effect (as has been found in other taxa ([e.g. see1 and references therein])), then the total effect size will be larger. A second issue is that females store sperm, and this sperm is getting older inside the female. Previously acquired sperm is not completely replaced by mating, which makes attempts to control male age independent of female age very difficult or impossible. In nearly all aspects of our experimental design we have had to make compromises to maximise power because the natural environment is so noisy in relation to fitness effects that we need to focus on a small number of manipulated factors and maximise our power to test these. We have added text to our methods section to explain exactly the issues that the reviewer raises, including the point about sperm age discussed above.

I'm also not sure how to interpret the statements on interaction effects in this study (e.g. P9 L20 - P10 L2; P11 L18-22). The authors appear to be saying, on *a priori* grounds, that they do not have enough power to reliably detect interactions. Does this mean that that they will not test for interactions in their data? Or that they will test for interactions, but then disregard interactions as unreliable even if some interactions are found to have statistical support? If the authors feel that they are unlikely to have sufficient statistical power to test for interactions then why use a factorial design? If interactions are likely to be present (even if undetectable statistically), then how can this study provide a reliable test of the main effects?

This is also an insightful comment, and another issue that we have agonised over. The reality is that, in common with the vast majority of studies on wild animals, our estimates of effect size are subject to enormous uncertainty. For the purpose of designing the study we have necessarily had to take a conservative view of likely effect sizes. With these conservative assumptions, to robustly identify interactions between inbreeding and parental age would require a study with sample sizes that will almost certainly never be possible. However, we will be able to provide some estimates of the effect size of interactions which will be very interesting. We have clarified this point in the ms in the 2^nd^ half of page 10 and onto page 11. We have also modified our power analysis to include a small interaction in the simulations. This analysis had to be re-done because the change in design to include laying date as a continuous variable rather than as a separate treatment group meant that we now have a larger sample size for our main treatments.

In relation to the concern that if there are interactions then this will prevent us from providing a reliable test of the main effects, the available evidence in relation to interactions between inbreeding and stress indicates that under natural conditions inbreeding consistently increases the impact of other stresses (such as parental age)[2, 3]. This indicates that if there are interactions, they will not mask the main effects that our study is designed to detect. There would only be a problem with the interactions if the two effects of higher inbreeding or greater parental age acted in opposition to one another, whereas all the evidence suggests that they will either act independently or multiplicatively. This means that implementing treatments orthogonally is not expected to decrease the power of our study in relation to either independent treatment. We discuss this point in the revised manuscript at the top of page 11.

Other comments:

P4 L24 - P5 L2: There are words missing. These sentences are unclear.

We have revised this paragraph extensively to clarify our meaning – apologies for the earlier errors.

P12 L6-8: How were the temperatures chosen? Are they ecologically relevant?

These temperatures are certainly within the range that crickets experience in nature. The natural environment involves temperatures many degrees higher and lower than these temperatures, however, in nature the temperature is constantly fluctuating. Temperatures below 18°C arrest development altogether in developing nymphs, but crickets survive well. Our pilot studies indicated that temperatures higher than 33°C lead to high mortality. In fact after submitting this ms we carried out further pilot studies and have found that mortality is still very high at 33°C, so we have revised our temperature plan to use 18°C and 28°C. These are temperatures that we used in a published study examining temperature-dependent differences in growth rate between crickets from high and low altitude populations[4].

P34 L7-9: There are indeed very few such studies, but there are some. For example, a study on rotifers investigated maternal age effects when mothers were maintained on normal vs. restricted diets (Gribble et al. 2014 Maternal caloric restriction partially rescues the deleterious effects of advanced maternal age on offspring Aging Cell 13, 623-630 ). There have also been a couple of studies on mice with manipulations of maternal diet and age at breeding. Perhaps the authors are referring specifically to the effects of temperature?

Yes, good point. We have re-written this sentence to be explicit about temperature and also to bring it more in line with the reviewer’s earlier point that manipulations of temperature are not necessarily *only* manipulations of physiological ageing rate.


Rev. 2:
There is a massive gap in our understanding of ageing in natural populations of invertebrates, and 'Impacts of parental age and inbreeding on fitness in a wild insect' proposes a much-needed investigation into this area. The manuscript is very well written: it is straightforward, complete, and interesting, and I found no substantive omissions or inaccuracies. The hypotheses are well-supported, and the experimental design is appropriate. I do have concerns that the 20 day-old crickets are truly "old" (see comments below). This choice needs to be justified because if they are not truly old, then the study may not actually be assessing a range of ages relevant to the phenomenon of senescence. Even if this choice of 20 days cannot be justified, a greater age might simply be substituted (although there may be feasibility concerns that I am not aware of).

As far as I can tell, the design is sufficiently powered (with the caveat above). The controls appear appropriate and well-considered.
I think that this could be a valuable contribution to the ecological study of ageing, and I would very much like to see this work carried out and published.

We appreciate the supportive comments. We have taken the feedback about the 20 day threshold very seriously and modified our proposed design to include a greater among-individual age effect as described below where this point is raised.

1. p2 (6) "time" is unclear. Is this 'age' or 'date'?
2. p2 (6-7): "so changes…are poorly understood". I do not follow this logic.
Yes – apologies we were trying to be clever with the text in these sentences at the expense of making sense, we have now revised to something that is hopefully much clearer.

3. p2 (10): "divide between field … and laboratory studies". This divide is not introduced prior to this.
Yes, good point, we have revised the previous sentence to introduce this point.

4. p3 (10): may be profoundly…

Yes, we were getting carried away there, we have rewritten the sentence to be more measured and precise.

5. p4 (10-11): Isn't the referenced paper [13] about juvenile survival rather than the Lansing Effect?

Yes, it is and we were clear about that (the text reads *“Many field studies have examined parental age effects on offspring traits other than longevity, most commonly survival of juveniles. Ivimey-Cook and Moorad [13] identified studies of maternal age effects on pre-adult survival in 97 species.*). We think these studies of maternal age effects on survival rather than longevity are nevertheless very relevant to the Lansing effect.

6. p4 (15-17): I can see how experimental manipulation could be helpful (for example, by generating/removing correlations between parental ages to improve statistical power to detect interaction effects), but I do not understand how this can ameliorate or better estimate the effects of selective disappearance.

We revised this sentence so that it now reads *“To make progress in this field, we need experimental studies in which the same parents are used at young and old ages.”* Hopefully this clarifies how such experiments can avoid selective disappearance.

7. p4 (24) to p5 (2): I do not follow this argument. Perhaps if the penultimate sentence (24-25 appears to be a fragment) was amended, it might become clearer.

Yes - apologies there was a fragment of a sentence here and the whole thing was not very clear. We have now revised this text so it reads: *“In annual species, selection for short generation time is absent; this removes the difficulty of comparing alternative ‘fast’ and ‘slow’ life-history strategies. Generation time strategies are difficult to compare because the selection benefits of short generations result from how fast alleles can multiply, but the costs are paid through reduced longevity or reproductive rate.”*


8. p5 (11-13): I have to admit that I am a bit sceptical that 'biological age' is a useful concept. Ignoring that, I cannot see how altering condition necessarily 'resolves' complications arising from selective disappearance. Within some environment, we can expect that observed rates of aging are the summed effects of longitudinal aging and selective disappearance. If we change the environment, we might change that balance, but I should think that the most likely outcome would be to change the magnitude of both factors in the same way (i.e., a more stressful environment would both accelerate longitudinal senescence and intensify the effects of selective disappearance). Working this out could be interesting, but I see this as manipulating the environment and assessing changes in ageing, rather than directly manipulating ageing. Also, is selective disappearance a problem or a phenomenon with studying in its own right? I would lean towards the latter given that we have
statistical methods that can help us measure its effects on many traits.

Reviewer 1 is also not a fan of the biological age concept, we liked it, but when both reviewers agree, this is strong evidence. Also we do have some sympathy with this point – biological age is used in various ways mainly in the human gerontology literature. We have revised the ms to remove this description throughout. We main now refer to an ‘environmental temperature treatment’ which is a much more agnostic description and we occasionally refer to ‘physiological age’ and have added text explaining how physiology is affected by temperature. We have also taken out the comments about resolving complications arising from selective disappearance. We agree that studying selective disappearance is very interesting, and we agree with the assessment that this isn’t what our study is doing. We have rewritten the text in this section so that we are more explicit about what we mean when we say that we are manipulating ageing. We think the claim that we’re manipulating ageing is sustainable, because it seems inevitable that ectotherms kept at high temperatures will senescence at a faster rate, so one could regard our environmental temperature treatment as a manipulation of physiological age. However, we have toned down this claim a lot.

9. p6 (24): remove comma

Done

10. p7 (8-10) : also see Yun and Agrawal (2014) for an interesting take on this...

Yes, we are aware of this work – we started adding a more detailed discussion of complex relationship between stress and inbreeding depression. However, to do this properly became rather a long section. Since we are not specifically targeting this interaction in our analyses (because we cannot reliably anticipate the effect size) we decided not to get into the discussion of stress and inbreeding depression interactions. There are complexities to this as revealed by Yun and Agrawal (2014)[5], but the general pattern is robust and even Yun and Agrawal (2014) do find increased inbreeding depression with stress, it’s just that they find that density dependence is a stronger factor.

11. p7 (12): "lab-as-field studies" - I do not know this term

We invented it; probably not a good idea. We have removed it and provided a more straightforward description.

12. p7 (end): The amount of ID experienced by a population follows from both the prevalence and degree of inbreeding and the effects of inbreeding when inbred. The potential for ID follows just from the latter. If it is true that you are assessing the latter, could you make this clear here?

Yes, this is a very good point, we have modified the text to make the distinction between inbreeding risk and costs of inbreeding, and to clarify that it is that latter we’re focussing on.

13. p8 (15): was this effect an increase or decrease?

We have clarified that these effects were negative. We did mention this in the subsequent sentence, but you’re right, it is easy to add the word ‘negative’ which is an improvement.

14. p9 (4-6): It is not clear to me why this necessarily leads to a weak design. Please explain.

Yes, this was a bit of a throw-away remark, that intended to contrast a within-subjects design with an among-subjects design. We think, on reflection we should just omit this sentence.

15. p9 (9-10) : Can we say with confidence that telomere attrition has a causal effect on offspring performance? If not, perhaps noting that the relationship is correlational would be safer.

We certainly can’t say this, and it wasn’t our intention to imply this – in the previous sentence we pointed out that there is no evidence for telomeres in relation to ageing in this species. We changed the text to try to make sure we’re not misunderstood.

16. p9 (19-20): is this directly relevant?

We included this (the line “*This possibility has not been directly studied, but interactions between parental and grandparental age are known* [6]”) because it illustrates that parental age effects may interact with other non-genetic effects. We have now added the phrase ‘*because it illustrates that parental age effects may interact with other non-genetic effects’* because as you point out, its relevance isn’t clear without this clarification.

17. p10 (9-11) : I do not follow this logic.

The reviewers’ comment refers to the statement: “*We know there are significant costs of inbreeding in crickets, so there is a clear prediction that outbred males will have greater mating success than inbred males. If inbred males do not do measurably worse than outbred males, it suggests that measuring mate choice in wild insects may be beyond the scope of all but extremely large studies*.” We have now rewritten the text around this statement to clarify our meaning.

18. p11 (12-22): I think that this section of the paragraph essentially repeats elements from the end of p.9. The text could be streamlined a little to save some length.

Yes this is a good point, we have reduced this paragraph and made the important point about interactions not reducing our power to test main effects in the previous section. As anticipated, this has saved about half of the text.

19. p12 (5): Can you justify representing 20 day-old crickets as "old"? I appreciate that this can be a bit subjective, but I think that this is important. One way to do this might be to refer to earlier work that finds senescent declines at this age. Another might be to present evidence that cumulative survival rates have appreciably declined by the age and suggest that this implies a substantial loss of selection to maintain performance. The latter is a gambit used by Ivimey Cook and Moorads' study of maternal age effects in N. vespilloides (AmNat 2018).

The concern about whether 20 days is enough is a serious point that we have also been agonising over. In our field populations, we do find senescent declines that begin at around 15 days post-adulthood [7]. The average adult lifespan is 28.9±17.9 days (mean±SD, N=1,135)[7] so 20 days is 2/3 of the way through the average adult life. However, we have observed crickets living up to 84 days in the wild[7]. On reflection we have come to the conclusion that a greater range of ages might be preferable. In response to this comment and comments by reviewer 1 we have revised our design (see also comments to reviewer 1, above). The within-individual effect is constrained to being around a 20 day difference between ‘young’ and ‘old’ individuals because our past experience shows that once females begin laying, many of them will not continue to lay for a lot more than 20 days, and we will then have a situation where the ‘old’ group are a non-random sample of the population.

In our modified design, we will create a greater range of ages of first matings and subsequent egg laying by mating females for the first time when they are from 10 days up to 40 days old (studies we ran this spring found that in the lab they are reluctant to mate immediately after becoming adult). This means that our old females will be much older, and effectively adds an among-individual age comparison to our within-individual age comparison. So, this modification means that we will have the same within-individual 20 day ageing treatment, but an additional among-individual ageing treatment with a range of parental ages of first reproduction. This range will not be confounded with the time in the season because crickets will become adult over range of dates. Because we will be mating crickets for the first time at a range of ages (rather than just having ‘young’ and (20 day) ‘old’ parents) it no longer makes sense to have a separate group that are mated when they are young but are made up of individuals that have recently emerged. In our new, simpler design, first mating individuals will include a range of parental ages and of dates in the season when eggs are laid, that will be orthogonal to one another. Using the statistical methods mentioned by the reviewer in their previous comment we will then be able to tease apart the effects of among and within individual age and test for selective disappearance.

20. p12 (10): This would be a daunting standard to be held to!

Yes, we agree! but figured it was better to directly address this point…

21. p12 (17): remove comma

Done

22. p12 (17-21): I take this to mean that because this species does not have overlapping generations, there is no a priori expectation that the timing of reproduction affects fitness. Is this correct?

Yes, this is correct, we have modified the text slightly (using the reviewers’ wording) to try to make this a bit clearer.

23. p20 (Table 2): Incidentally, if these are aligned in the direction of higher fitness, than they correspond to directional selection gradients.

Yes, that’s true, we have swapped the alignment and made this point in the table legend.

24. p20 (3): Given that you have established that generations are non-overlapping, you can justify calling this 'fitness'. I think that this is worth pointing out since few wild animal studies can make this claim.

Always a touchy subject, but we have added this point to the text…

25. p21 (4-6): Does this average take into the account of censoring? Also, how does your assessment of 20-day-olds as being 'old' hold up given these estimates?

This is the mean age of death so it is not adjusted for censoring. We have now moved these stats to earlier in the ms as part of our general revision to accept the reviewer’s point about 20 days not being old enough. We also added and referenced our earlier finding of senescent declines being present from 15 days old onwards.

26. p21 (11): bracket is opened but not closed

Done

27. p21 (14-16): repeated from before?

Yes, it was thanks – we have deleted this instance.

28. p21 (18): and we will move

Done (we opted for a semi-colon).

29. p22 (6): remove comma

Done – we rejigged the text somewhat as it was a bit clunky in general.


30. Study Design Table: I do not feel strongly about this, and but it seems to me that most of this table is superfluous because it largely repeats what is in the text (an exception is the explicit models that are fit in the 'Analysis Plan' column). I would urge the authors to consider whether this table is necessary.

We are not big fans of the study design table either, and we tried to avoid including it. However, this is a requirement of the PLOS format and we can see merit in having certain compulsory elements for this type of submission.

1.	Wylde Z, Spagopoulou F, Hooper AK, Maklakov AA, Bonduriansky R. Parental breeding age effects on descendants’ longevity interact over 2 generations in matrilines and patrilines. PLOS Biology. 2019;17(11):e3000556. doi: 10.1371/journal.pbio.3000556.

2.	Richardson J, Smiseth PT. Chapter Two - A behavioral ecology perspective on inbreeding and inbreeding depression. In: Podos J, Healy S, editors. Advances in the Study of Behavior. 55: Academic Press; 2023. p. 37-54.

3.	Armbruster P, Reed DH. Inbreeding depression in benign and stressful environments. Heredity. 2005;95(3):235-42. doi: 10.1038/sj.hdy.6800721.

4.	Tregenza T, Rodríguez-Muñoz R, Boonekamp Jelle J, Hopwood P, E., Sørensen J, G., Bechsgaard J, et al. Evidence for genetic isolation and local adaptation in the field cricket *Gryllus campestris*. J Evol Biol. 2021;34:1624-36. doi: 10.1111/jeb.13911.

5.	Yun L, Agrawal AF. Variation in the strength of inbreeding depression across environments: effects of stress and density dependence. Evolution. 2014;68(12):3599-606. Epub 2014/09/13. doi: 10.1111/evo.12527. PubMed PMID: 25213285.

6.	Promislow DEL, Flatt T, Bonduriansky R. The biology of aging in insects: form *Drosophila* to other insects and back. Annu Rev Entomol. 2022;67(1):83-103. doi: 10.1146/annurev-ento-061621-064341.

7.	Rodríguez-Muñoz R, Boonekamp JJ, Liu XP, Skicko I, Haugland Pedersen S, Fisher DN, et al. Comparing individual and population measures of senescence across 10 years in a wild insect population. Evolution. 2019;73(0):293-302. doi: 10.1111/evo.13674.
