# Supplementary material for: Impacts of parental age and inbreeding on fitness in a wild insect
Source: PLoS Biol. 2026 Mar 10;24(3):e3003713. doi: 10.1371/journal.pbio.3003713 (PMC12991364; doi:10.1371/journal.pbio.3003713)
Supplement: Attachment — Submitted filename: PBIOLOGY-D-23-00686 AE comments.docx [file pbio.3003713.s003.docx]

I have now read the revised pre-registration and the responses to the reviewers’ comments. It general it seems fine. I have no problems with the Introduction. There are, however, still a few places in the Methods and Study Design Table where I was unsure about the design/analysis. This might simply be due to a lack of clarity on the part of the authors, or maybe I simply misread what they wrote. For what it is worth, these are:

Fig 1. Clarify that for the part of the figure shown below, that pairings A1A1 to B4B4 have parents that then go into the cold treatment and A5A5 to B8B8 go into the cold treatment. Easy to show this by splitting the orange box in half: left cool, right hot.

Fig 1 -  Is the criteria for survival of nymphs to retain a box greater than 20 or 30 surviving - both are shown (see image below).

Fig 1 - What is the expected success rate of boxes? The text refers to 70% but 40 of 48 is 83%. Obviously this Is beyond the authors’ control but it’s confusing to not be precise here.

Fig 1 - Is it 40 or 60 boxes? Both numbers are given. I assume 40. You could also clarify each of these 40 are then split so that EACH location has 40 boxes. The phrase ’split the boxes’ can be read in two ways: 20 & 20 or 40 x 2. I know this is trivial, but it takes detective work to figure this out as a reader.

For the above corrections, check that the text matches the figures (e.g page 14 refers to 60 boxes).

Finally, the stats model are not that clear to me. Again, just adding a few clarifying remarks would save the reader having to piece together the text and figures/tables.

1. To test inbreeding effects, you use this model

Can you confirm this means that you are ignoring the effect of temperature because you only use first ‘broods’ for this analysis? I assume so given there is no term in the mode for first vs second ‘brood’. If so, does this makes sense? Aren’t you losing information on the effect of inbreeding by ignoring the second broods?

2. For the effect of temperature, you use this model

Can you confirm that this model is only using data from second broods? I assume so given there is no factor to distinguish first vs second brood*. Can you also clarify that ‘laydate’ only refers to the first brood (and does ‘median’ refer to the fact that eggs are laid over several days?). Will this model be run separately for inbred and outbred crickets (as there is no factor for inbred/outbred)? If so, isn’t this a missed opportunity to test for an interaction between inbreeding status and the temp effect?

* I don’t think age2 works if that is the intention and you are including first and second broods, unless you intend to include 0 as the value for first broods (in which case you need to have a random factor for pairID). If I have all this muddle up then I think the onus is on the authors to be clearer about the planned stats.

I hope these comments are helpful.
