# Supplementary material for: Impacts of parental age and inbreeding on fitness in a wild insect
Source: PLoS Biol. 2026 Mar 10;24(3):e3003713. doi: 10.1371/journal.pbio.3003713 (PMC12991364; doi:10.1371/journal.pbio.3003713)
Supplement: Attachment — Submitted filename: PLOSBIO notes for reviewers stage2.docx [file pbio.3003713.s006.docx]

## PLOS Biology PBIOLOGY-D-23-00686R3 Stage 2 submission notes for reviewers

As requested, this document details changes made to the manuscript since stage 1 acceptance. In line with the concept of the pre-registered report we limited changes to the document following stage 1 acceptance as much as possible. We have changed the tense of various aspects of the introduction and the methods so that the piece reads like a normal paper. Otherwise we have sought to avoid changes. However, one aspect of our experimental protocol did not function as we were expecting, and this necessitated changes to the introduction and the methods to avoid them being very cryptic for readers not familiar with the history of the project. We are entirely transparent about these changes and how our final dataset differs from the one we anticipated we would have, but we have made some changes to avoid the methods (in particular) being very confusing for readers who have not read the pre-registered report.

Specifically (and as described in the Stage 2 ms) our plan was to collect eggs from females at young age and then isolate them for 20 days at one of two temperatures and then collect eggs from them again 20 days later. However, we found that although females laid eggs across a wide range of initial ages (from 6 to 32 days post adulthood), unexpectedly, the vast majority of the eggs from the second age period did not hatch: only two females produced offspring from this second stage of egg collection. This meant that although the inbred/outbred aspect of our design was executed exactly as planned and we also had a very powerful dataset providing an among-individual study of the effect of parental age, we could not compare the effect of parental age within-individuals.

**Description of changes by section:**

*Abstract*

Only changes are to the tense and an additional 3 sentences at the end of the abstract which describe our results and provide a sentence of discussion

*Introduction*

We took out two sentences at the beginning of the introduction which were largely a pitch to support the execution of the project but which don’t really add much, now that we have carried out the work. These sentences were:

*“Our aim is to examine the impact on fitness in nature of two population-ecology dependent parameters: inbreeding and parental age. Both these factors have been shown to have substantial impacts on fitness in a range of animals studied in the lab. However, despite, their potential to occur in almost any species and their likely sensitivity to environmental change, we have almost no idea about how important these factors are in natural populations.”.*

We also removed 3 sentences that described the rationale for an aspect of our design which we were forced to modify as described above. These sentences were:

*“We will examine broad effects of age and experimentally manipulate environmental temperature during aging. By manipulating environmental temperature, we will produce individuals that have lived for the same length of time, but have experienced different temperature regimes, so could be regarded as physiologically being at different ages [15, 16]. This allows us to isolate endogenous declines which will be dependent on metabolic rate (and hence temperature), from environmental impacts on organisms that accumulate as time passes, irrespective of temperature.”*

We have retained a full description of how we attempted to do this in the methods (exactly as we had committed to). However, since eggs laid after this temperature treatment failed to hatch, including this in the introduction is likely to be confusing for readers.

*Methods*

We added an opening sentence explaining that our study follows a design that was pre-registered with PLOS Biology and the Open Science Foundation in 2023 [53].

We retained an explanation of our full design which is summarised in the schematic in Figure 1 which is unchanged from our original submission.

We followed the analytical plan set out in our study design table

However, because although the inbred/outbred aspect of our design was executed exactly as planned, and we also had a very powerful dataset in relation to an among-individual study of the effect of parental age, we could not compare the effect of age within-individuals. For this reason, we removed Table 1, which was in our pre-registered report because its description of treatments in our study was no longer representative and will be very confusing for nearly all readers. We also removed the figures showing photographs of crickets next to a burrow and the rearing boxes in the meadow (previously figures 2 and 3) because these were not strictly necessary (we can reinstate them if desired). In the previous version we stated that “Both sexes excavate burrows in the late summer” we have modified this to “within a few weeks of hatching” because studies we have made in the meantime revealed this earlier burrow digging behaviour. This has no implications for our study, but it is useful background information.

We have removed the pre-registration specific components such as the study design table, power analyses, timeline and pilot data sections.

We have written the new results and discussion sections and we have deposited our data with FigShare (link to follow).
