# Supplementary material for: Impacts of parental age and inbreeding on fitness in a wild insect
Source: PLoS Biol. 2026 Mar 10;24(3):e3003713. doi: 10.1371/journal.pbio.3003713 (PMC12991364; doi:10.1371/journal.pbio.3003713)
Supplement: Attachment — Submitted filename: PLOSBIO stage2 response to reviewers.docx [file pbio.3003713.s007.docx]

Rev. 1:

This pre-registered study investigated effects of parental age, inbreeding, and ambient temperature on the fitness of female and male offspring in the wild. As the authors note, few studies have tested such effects in wild insects, which makes this study quite valuable. The results are somewhat difficult to interpret, which is not surprising for a field study. Nonetheless, I have some concerns about the analysis and interpretation. I also believe that the presentation could be improved.

Major comments:

The manuscript accurately describes the pre-registered plan. However, in terms of presentation, **the Introduction and Methods sections are very long, wordy and quite repetitive in places, making the manuscript difficult to read and understand.** The Introduction could perhaps be streamlined to avoid jumping back and forth between topics. It could also be shortened quite a bit. The Methods section repeats the same information in several places (e.g. L281-284). The long outline of the unsuccessful plan to measure offspring produced by the same females from eggs laid 20 days after the initial egg collection could be cut. Just explain briefly that within-individual comparison was not possible as a result of hatching failure of the late-life eggs.

The editor has confirmed that it is a condition of the PRR format that we do not change the introduction, so we have not done this. We agree with the point that the aspects of the methods which no longer directly relate to the results add a length to the methods which most readers will not be interested in. We have modified our methods by retaining the original plan (Fig.1), but indicating where the hatching failure of the late-life eggs meant that we changed our approach and focussed on the among individual variation in parental age which was also part of the original plan.

**I was surprised by the authors' conclusion that parental age at breeding had little or no effect on offspring performance.** Almost complete failure of eggs from the late-life egg collection to hatch could be interpreted as a very strong, negative parental age effect on offspring viability. It appears that the effects of advanced parental age were highly deleterious for embryos, resulting in the death of nearly all late-life eggs. Many insects continue to lay eggs at advanced ages when almost none of the eggs are viable. Perhaps these crickets were doing the same. How else could this hatching failure be interpreted?

We made it clear in our pre-registered report that this study would examine the effects of parental age and inbreeding on **adult** offspring. Our conclusions are about adult offspring. We do have data on eggs and juveniles in the lab which we are planning to analyse and which will form another ms which we have added reference to in the revision of this manuscript [1]. We deliberately chose not to include this additional study in the present ms because it would turn this already long ms into a huge tome making it difficult to digest in terms of all sections of the ms. In this ms, we refer to the effects of our treatments on eggs and nymphs in the context of how they affect our interpretation of the effects on adults. However, including these data would be a complete departure from our preregistered plan, and would require a completely new introduction and methods section.

The authors do acknowledge and discuss the potential effect of hatching failure in the context of inbreeding, and acknowledge that much lower survival of inbred offspring could have biased the sample of adults. A potential mechanism for this that's perhaps worth mentioning more explicitly is that individuals homozygous for strongly deleterious recessive alleles would have died as embryos, resulting in an adult sample consisting of individuals that did not express such alleles. Another potential reason for the lack of inbreeding effects on adults could be that the source population had recently experienced a bottleneck. In such a case, many deleterious recessive alleles would likely have been purged in previous generations.

Yes, we agree with the reviewer about this; we touch on this point in the discussion, however, since these data cannot be included in this manuscript, we have not gone into this interesting discussion of what might drive these effects.

The authors housed juvenile crickets at high and low altitudes, but did not include altitude as a treatment in their analysis. This seems problematic because altitude appears to have affected both nymph survival and adult body size. I think altitude should be included in the models for the various fitness-related traits.

We did include altitude in our analysis as we said we’d do in the PRR, it is the term “Envj” in our models and “Environment Low” in the tables. We have made it clearer in the methods section (first paragraph under “data analysis”) that we have done this in the revised document.

Other:

L220: Why "additionally"?

Because the two aims: to study the effect of inbreeding and the effect of parental age are largely independent points. By doing both things we could examine interactions between them, but the main motivation for doing both things was that this study took many person-years of work and we could run both studies simultaneously using our design providing an investigation into two important hypotheses in evolutionary ecology.

When there were not enough cameras for every active burrow, how did you choose which burrows would have cameras?

We moved cameras to try to provide information on as large a proportion of the adult population as possible, we think this is captured by the line *“we moved cameras around the meadow every few days, we were able to capture a large proportion of matings as well as to identify most of the pairings”.* We could expand this explanation further but it would make the methods longer, and isn’t easy to justify in the context of the methods being set out in advance.

For Fig. 3 and Fig. 4, please explain what the points and whiskers mean. I guess this is mean +/- SE?

Good point. We have now added that information to the figure legends. What we represent is actually mean ± 95% confidence intervals obtained from the *emmeans* R package. We think that confidence intervals are easier for readers to intuitively understand that standard errors.

Rev. 2:

SUMMARY

This study represents a valuable contribution to understanding inbreeding and parental age effects in wild insects, a research area where field studies are exceptionally rare. The authors deserve credit for undertaking the substantial logistical challenge of conducting experimental manipulations in a natural population and for pre-registering their study.

However, the manuscript requires substantial revision. The primary issues are: (1) overly rigid adherence to pre-registration format that obscures what was accomplished, (2) missing parental longevity covariate (standard analytical approach), (3) inadequate justification that the age range includes senescent individuals, (4) design limitation (confounded maternal/paternal age) not properly addressed, and (5) weak conceptual development (no clear predictions despite available theory).

Some of these points are quite difficult in the context of this being a preregistered report: our analytical approach was thoroughly reviewed over several rounds. The reviewers’ points above are made again below and we address them there.

The manuscript needs restructuring to clearly present the study conducted (cross-sectional age variation plus experimental inbreeding) rather than remaining constrained by pre-registered components that failed (within-individual age comparison, temperature manipulation). **With appropriate revisions, this could be an important contribution.**

Recommendation: Major revisions required. Given the fundamental nature of some issues, I recommend substantial revision before detailed line-by-line review.

We have consulted the Editor who has clarified that the conditions of the pre-registered report are that we cannot change the introduction and modifications to the methods need to be thoroughly justified. We have made some modification to the methods as described in our response to reviewer 1 above and below.

OVERARCHING ISSUE: Overly Rigid Application of Pre-Registration The authors treat pre-registration as a constraint on clear presentation rather than a commitment to transparency. The temperature manipulation and within-individual age comparison produced no data (eggs didn't hatch), yet these are described extensively in Methods, illustrated in Figure 1 ("unchanged from original submission"), and discussed as if relevant. This obscures what was actually measured.

We share some of the reviewer’s frustration over this point; at the same time we completely understand the editor’s point that not changing the introduction is a major part of the motivation for pre-registration and we have followed instructions on this. It is a not fair to berate us for treating preregistration as a constraint on clarity: we have been told what the rules are and we are following them.

The Methods describe analyses as "the approach described in the first half of our design table" rather than simply describing what was done, making the section unnecessarily difficult to follow.

Yes, fair enough, most readers won’t bother to refer to that table, the comment was designed to reassure readers that we have followed the preregistered plan. However, we accept the reviewer’s point that we sacrifice too much readability over this issue, so we have revised the text here.

Pre-adult survival shows a 40% inbreeding effect (larger than any adult effect) but is relegated to another paper because it "was not part of our pre-registered plan." However, survival to adulthood is necessarily measured and well-known to be affected by inbreeding. This should have been anticipated.

As discussed above, the effects of inbreeding on eggs and nymphs is a major study in its own right. Our preregistered article was always about adults. We cannot simply add these results here; they would require pages of new introduction and methods with all the relevant details about how these data were collected. It was partly to avoid this type of post-hoc request for a different study that we preregistered this study.

In my view, pre-registration should commit to publishing results regardless of outcomes while allowing clear, complete presentation. **It should not require extensive description of failed experiments**, prohibit reporting important patterns in necessarily collected data, or force confusing organization.

Required: Restructure to clearly present the study conducted. Briefly acknowledge what failed (one Methods paragraph suffices). Report all major patterns including pre-adult survival. Organize for reader comprehension. Present Introduction and Discussion matching the realized design.

The reviewer is very frustrated with the PRR format. We have addressed these points above.

CRITICAL ISSUES

1. Missing parental longevity covariate

The authors maintained parents until death but did NOT include parental longevity as a covariate. This is standard practice for controlling selective disappearance and separating aging from quality effects, appropriate even in the absence of longitudinal data (van de Pol and Verhulst 2006; Ivimey-Cook and Moorad 2020, both cited). Even with only 10% mortality by age 39, quality heterogeneity among survivors (reflected in eventual longevity) could mask age effects. Parents who reproduced at age 30 and died at 40 days differ fundamentally from those who reproduced at 30 and died at 70 days.

Required: Re-analyze including parental longevity as a covariate (continuous or categorical). Report whether age effects remain null after controlling for parent quality.

We explained that pre-reproductive mortality in the parental generation was very low <10% died earlier than the oldest reproducing crickets in the entire cohort (mainly only a few days earlier). For this mortality to obscure an effect of parental age would require both large variation in heritable traits affecting offspring performance and a large covariation between these traits and lifespan in parents. To address the reviewer’s concern, we have run the analyses with parental lifespan (for both parents) included as a covariate as suggested by the reviewer. These models (shown at the end of this document) confirm that the inclusion of parental lifespan does not significantly affect the estimates of parental age on any of the offspring traits we investigated.

This confirms our interpretation that the influence of selective disappearance is virtually non-existent. Furthermore, none of the estimates for parental lifespan reached significance, suggesting that parental lifespan is not predictive for any of our offspring traits. Given that parental lifespan was not included in our stage 1 manuscript and that we already address the issue in our discussion we do not feel that the reviewer comment warrants this deviation from the previously agreed statistical approach. If the editor would like us to add these additional analyses to a supporting information document, possibly in a section entitled “Post-hoc analyses suggested by reviewers”. we could do that, but we do not believe that many readers will be worrying about this possibility.

2. No justification that age range includes senescent individuals My Stage 1 Comment #19 asked for justification that the age range includes "old" individuals, suggesting survival curves or evidence of senescent declines at these ages. The authors cite "senescent declines" without specifying ages and state mean lifespan is 29 days. However, only 19/196 parents (10%) died by age 39. If 90% survive to age 39, calling 39-day-olds "old" is questionable. The age range may span young to middle-aged adults, not young to senescent.

This matters because null results could reflect true absence of effects OR insufficient age range that doesn't actually test senescent reproduction.

Required: Provide survival curves for this population. Specify ages at which senescent declines occur in cited studies. Explicitly justify that age range 6-39 days includes biologically "old" individuals. Reconcile 10% mortality by age 39 with mean lifespan of 29 days. If evidence cannot be provided, acknowledge this as a major limitation.

It is a fair point that readers may not believe that senescence occurs on this timescale even though it is much longer than the average lifespan of wild crickets. The It is also worth pointing out that senescence is not the same as death. Senescence typically begins soon after maturity (hence, in humans, the lack of 40 year old sprinters); lifespan in very benign conditions (e.g. the lab) is not a good measure of senescence. Fortunately, we have already published several studies which feature exactly the type of survival curves the reviewer requests [2-4]. These studies (in the same population of crickets as this ms) (e.g. see Fig. 2 in [2] for actuarial senescence and Fig. 1. in [3] for phenotypic senescence). These studies demonstrate that senescent declines that occur well within the range of ages that we used as parents in this study. We have added the information about the age of onset of senescent declines and the relevant references to our methods section. The apparent discrepancy between 10% mortality by age 39 with mean lifespan of 29 days is because the 29 days is lifespan in the wild, whereas the 10% mortality by age 39 is crickets kept in a benign laboratory environment. The size of this discrepancy is an illustration of why we need studies of insects in nature.

3. Incorrect statement about cross-sectional studies The text states "previous cross-sectional studies may severely underestimate the effects of parental age on offspring quality," implying ALL such studies are inherently flawed. However, van de Pol and Verhulst (2006), which the authors cite, shows cross-sectional studies CAN properly estimate aging effects if individual longevity is included as a covariate. The authors then conduct a cross-sectional study without using this solution.

Required: Revise to specify that cross-sectional studies WITHOUT longevity covariates may underestimate effects. Acknowledge the current study is cross-sectional and apply the longevity covariate approach.

We agree with the reviewer that the statement in the introduction could be read as applying to all longitudinal studies. This was not our intention; we are well aware of the issues around selective disappearance. We have applied van de Pol and Verhulst’s method extensively and published previous ageing studies with Verhulst [5, 6]. We are unable to change the introduction; however, the sentence is unclear rather than incorrect.

4. Contradiction about defining "old"

The Introduction argues for "experimental studies in which selective disappearance can be ruled out (i.e. there is no significant parental mortality until after the oldest parents have reproduced)." This is contradictory: if there's no significant mortality by when "oldest" parents reproduce, **those individuals aren't experiencing senescence** (characterized by elevated mortality). One cannot claim both that minimal mortality validates the design **AND that the study examines senescent parents.**

Required: Reconcile this contradiction. Provide survival curves showing when senescence begins. If the age range doesn't include senescent individuals, acknowledge you're not testing parental aging effects.

We categorically disagree with the statements above. The reviewer is talking about actuarial senescence (increased probability of mortality with age). However, in this study we are dealing with phenotypic senescence (declines in physiological performance with age). Phenotypic senescence is the really interesting biological phenomenon; actuarial senescence is a symptom of phenotypic senescence. Organisms experience senescence without dying, indeed two individuals with identical lifespans can differ in their degree of phenotypic senescence. Phenotypic senescence is the cause of the Lansing effect: if individuals have to die to be senescent then they could not reproduce. We have demonstrated phenotypic senescence in this species [2-4, 7] and we cite this work.

5. Figure 1 shows failed design

Figure 1 ("unchanged from original submission") shows temperature manipulation, second egg collection, and within-individual comparison. None produced data. Readers will misunderstand this as a within-individual experimental manipulation with temperature effects. The actual study is cross-sectional natural age variation plus inbreeding manipulation at a single time point.

Required: Revise Figure 1 to show the realized design or create two panels ("Planned" and "Realized") so readers understand what produced the data.

We agree with the reviewer that the presentation can lead to confusions. Though we carried out our study exactly as per agreed study design, eggs from the 2^nd^ phase of the study failed to hatch. We have added a statement at the start of the methods to make this clear and we have also highlighted it in the figure, which otherwise remains unchanged, to make clear which part was “realized”, as per reviewer’s suggestion (we use a hatched area, rather than creating two panels which turned out to be very hard to read). It is unnecessarily harsh to repeatedly refer to the design as ‘failed’, if we had not preregistered this study we could merrily gloss over this issue and readers would miss out insights that could be very useful in designing future studies etc.

6. Confounded maternal and paternal age

Maternal and paternal age are correlated (tau = 0.53) because pairs were kept together. The authors assert this creates "high collinearity" justifying separate models. However, **tau = 0.53 is moderate correlation**, not extreme collinearity preventing estimation. Critically, the authors don't report attempting a full model with both ages. Incidentally, I don't think that tau is necessarily the most appropriate measure: Pearson's r tells you about the linear relationship between the variables, which is exactly what matters for collinearity in linear regression.

The separate models misleadingly suggest distinct, independently tested effects. Each still estimates a confounded effect. The design was avoidable (could have paired young males with old females and vice versa).

Required: Run a model including both maternal and paternal age. If it converges with stable estimates, report it (allows testing whether effects differ). If it fails, report this and acknowledge the design cannot distinguish maternal from paternal effects. Reframe results accordingly. Remove "unsurprisingly" when describing the correlation, which obscures that it results from design choice.

These are sensible points about tau and a Pearson’s correlation. It turns out that this issue is resolved by the following: In our PRR we said that we would include parental age in our models, however, when we were analysing our results, we lost track of exactly how we said we would do it and used male and female age separately which created our concerns over collinearity. Returning to the table in our PRR, we clearly stated that we would use “Median parent chronological age when the first batch of eggs were laid” (see explanation of terms at the end of the analysis table). This approach was in all the reviewed version of the document, and we believe it makes sense to use this approach (as well as it being what we planned to do). We have modified our submission accordingly. This does not change any of our qualitative findings. For the interest of the reviewer, we also ran a single model with both maternal and paternal age included as separate factors which gave the same results as the model in the resubmission. Interested readers will be able to run any model they wish using our deposited data.

MAJOR ISSUES

7. No predictions despite available theory The Discussion states "We did not have a strong prediction in relation to effects of parental age." This is problematic given: (1) evolutionary theory (Moorad and Nussey PNAS 2015 and others) predicts negative effects because selection weakens on traits expressed in offspring of older parents, (2) physiological mechanisms (mutation accumulation, oxidative damage, epigenetic changes) predict negative effects, (3) empirical data from G. bimaculatus (cited in Introduction) shows negative effects of parental age, (4) comparative data (Ivimey-Cook and Moorad 2020) shows 106 insect studies mostly finding negative effects, and (5) the authors made clear predictions for inbreeding based on similar logic.

Re: "Strong" predictions. Either you have a prediction or you don't. This suggests weak engagement with theory and makes the study appear exploratory rather than hypothesis-driven.

Required: State clear predictions based on theory and comparative data, then discuss null results in that context. Or explicitly justify why this species should differ from theoretical predictions and related species.

We agree with the reviewer that the sentence “We did not have a strong prediction...” was unhelpful, and we have removed the sentence from our discussion. The relevant literature and clear theoretical predictions were already discussed in the introduction. In the discussion we put our results in this context without repeating the same points as in the introduction. We also raise the issue pertaining the risk of publication bias in this subject area. Indeed, the paper by Ivimey-Cook and Moorad flagged critical study design issues in studies on the Lansing effect, which in part motivated us to preregister our study design. In the revised manuscript we have revised the discussion to make this clearer.

8. Interaction justification overstated

The text states the design "has allowed us to examine potential interactions." However, the pre-registration acknowledged interactions were "not a core aim" due to insufficient power. No interactions were found.

Required: Present this as two parallel investigations examined together for logistical efficiency, not as a study motivated by interaction testing.

Yes, this is a fair and constructive appraisal, we have modified the text as suggested.

9. Mismatch between motivation and measurement (Stage 1 Comment #12 inadequately addressed) Stage 1 Comment #12 asked for clarity about what the study measures. The Introduction states "We are desperately short of information about how great the risk of inbreeding is in wild insects, and about its potential to depress population viability," invoking population-level concerns. However, the study does not measure inbreeding prevalence (how often it occurs) or population-level risk (prevalence times severity). It experimentally creates full-sib matings to measure severity of inbreeding depression when it occurs.

Required: Revise the Introduction to clearly frame the study as measuring the cost or severity of inbreeding depression when it occurs in this species, which is one component (but not the only component) of assessing population-level risk.

We can see why the reviewer is annoyed about this, and in retrospect this sentence in the introduction could be more focussed on exactly what we study. However there are unlikely to be many readers that are actually going to be in any doubt what our study does. We are unable to change the introduction, but we don’t think that this point is a serious problem.

10. Overly complex explanations

The explanation of why annual species are useful remains convoluted despite Stage 1 Comment #22. The key point is simple: in species where generations don't overlap, fitness equals lifetime reproductive success. The current explanation involving "population growth rate parameters" obscures this. Similarly, the discussion of "alternative life-history strategies" versus "selective disappearance" is confusing because they appear to describe the same phenomenon (quality heterogeneity among individuals).

Required: Simplify these explanations.

Although this species is annual, fitness does not equal reproductive success. Population size in these crickets varies by as much as an order of magnitude among years [8]. In terms of the proportion of alleles that individuals contribute to future generations (fitness) this means that having 2 offspring in one year can be equivalent to having 20 offspring in another year. Our ‘convoluted’ description was an attempt to explain this point in response to Stage 1 Comment #22. We think that this is an important point, although the reviewer clearly disagrees. Mercifully in this case, we are bound to keep the introduction as it was when the ms was reviewed over several rounds.

MODERATE ISSUES

Methods organization: Study system description should precede experimental design. Readers need biological context before encountering protocols.

This was the case in our PRR, the methods are written so that they make sense without knowing the details of the study system which is presumably why the reviewers didn’t make this point at the time. We are happy to restructure the methods if the editor would like us to do this although it doesn’t appear in line with the PRR.

Evidence for senescence not used: The authors cite evidence for senescent declines but don't specify at what ages, missing an opportunity to justify their age range.

As per comments above, we have now cited our work in this area which has a lot of detail about this issues.

Paragraph breaks missing: New topics begin without breaks in several places.

Thank you, we have added some more paragraph breaks.

"WildCrickets" undefined: This term appears without explanation.

Thank you, we have added a reference to our website which provides a lot of details where we first use the term.

Terminology: "Standard normal standardization" should be "standardized to mean 0 and SD 1" with brief explanation of why.

Yes, good idea, we have changed the explanation to the wording suggested and given a brief explanation.

Distribution families not justified: Authors assert choices are "most appropriate" without explaining why (e.g., why negative binomial over Poisson for counts? Was overdispersion tested?).

Pre-adult survival deferred: The 40% survival reduction is larger than any adult effect but is relegated to another paper. While detailed age-specific analysis might warrant separate treatment, basic survival-to-adulthood statistics should be reported here as this is a fundamental fitness component necessarily measured.

We have addressed this point above – we can’t report “survival-to-adulthood statistics” without giving the details of the rearing regime etc. see earlier responses to this point.

STAGE 1 OVERSIGHTS

Several issues should have been caught at Stage 1:

Confounded maternal/paternal age: I failed to identify this design flaw. The design could have created independent variation by pairing young males with old females and vice versa. This cannot be fully fixed now but can potentially be salvaged by running the full model including both ages. If that fails, the limitation must be acknowledged.

We have addressed this point above

Weak dual-factor justification: The conceptual motivation for studying both inbreeding and parental age together needed clearer development, especially given that interaction testing was not a core aim.

We have addressed this point above – it is not really a major issue in terms of what readers will take from the study: researchers will use it for the information it provides about effects of inbreeding and parental age in the wild, they will not be preoccupied about our conceptual motivation which we have now set out even more clearly.

Pre-adult survival not pre-registered: Survival to adulthood is necessarily measured and well-known to be affected by inbreeding. This fundamental fitness component should have been included in pre-registered outcomes.

We did not include this because it is a huge enterprise in its own right, involved different people and would require a much expanded introduction and methods sections which would make the paper extremely long and unwieldy. We pre-registered this work partly to avoid reviewers demanding large scale post-hoc changes like this.

CONCLUSION

This represents valuable work in a challenging system where field studies are rare. With substantial revision addressing the critical issues (**particularly adding parental longevity covariate, justifying age range, attempting full model for maternal/paternal age, engaging with theory, and reporting pre-adult survival),** this could be an important contribution to understanding inbreeding and parental age effects in wild insects. The null results for parental age would be particularly interesting if properly justified and interpreted.

I am willing to provide detailed line-by-line review of a revised manuscript once these fundamental issues are addressed.

1.	García FR, Menes JP, Tregenza T, Boonekamp Jelle J, Rodríguez-Muñoz R, Ojanguren AF. The influence of inbreeding and parental age on preadult survival in the field cricket *Gryllus campestris*. in prep.

2.	Rodríguez-Muñoz R, Boonekamp Jelle J, Fisher D, Hopwood P, Tregenza T. Slower senescence in a wild insect population in years with a more female-biased sex ratio. Proc Roy Soc B. 2019;286(1900):20190286. doi: 10.1098/rspb.2019.0286.

3.	Rodríguez-Muñoz R, Boonekamp JJ, Liu XP, Skicko I, Fisher DN, Hopwood P, et al. Testing the effect of early-life reproductive effort on age-related decline in a wild insect. Evolution. 2019;73:317-28. doi: 10.1111/evo.13679.

4.	Rodríguez-Muñoz R, Boonekamp JJ, Liu XP, Skicko I, Haugland Pedersen S, Fisher DN, et al. Comparing individual and population measures of senescence across 10 years in a wild insect population. Evolution. 2019;73(0):293-302. doi: 10.1111/evo.13674.

5.	Boonekamp JJ, Salomons M, Bouwhuis S, Dijkstra C, Verhulst S. Reproductive effort accelerates actuarial senescence in wild birds: an experimental study. Ecol Lett. 2014;17(5):599-605. doi: 10.1111/ele.12263.

6.	Boonekamp J, Rodríguez-Muñoz R, Hopwood P, Zuidersma E, Mulder E, Wilson A, et al. Telomere length is highly heritable and independent of growth rate manipulated by temperature in field crickets. bioRxiv. 2020:2020.05.29.123216. doi: 10.1101/2020.05.29.123216.

7.	Makai G, Rodríguez-Muñoz R, Boonekamp Jelle J, Hopwood PE, Tregenza T. Males and females differ in how their behaviour changes with age in wild crickets. Anim Behav. 2020;164:1-8. doi: 10.1016/j.anbehav.2020.03.011.

8.	Rodríguez-Muñoz R, Hopwood P, Slate J, Walling C, Houslay TM, Tregenza T. Fluctuating selection among years in a wild insect. Evolution. 2025;79:973–82. doi: 10.1093/evolut/qpaf042.

Appendix: MODELS INCLUDING LIFESPAN FOR BOTH PARENTS

Models (for each sex separately), as in the main text. The sex is included in the name of the model right before the termination as “mod21”; F or M for female or male offspring. The name of each variable is at the beginning of the name. A brief explanation on how the variables are coded:

TW: Thorax width

Mass: Body mass

LS: Lifespan

Mates: Number of mates (for females only)

Mated: Whether the male mated or not (for males only)

MatedPortion: The proportion of matings a male got (only for mated males)

Calling: How frequently a male called

Won: Success in fights
